# 2766. "A Retrospective Analysis of Non-Blood Sources of *Achromobacter* Infection and Antimicrobial Resistance Patterns: A Mayo Clinic Enterprise Experience"

**DOI:** 10.1093/ofid/ofad500.2377

**Published:** 2023-11-27

**Authors:** Mitchell Dumais, Nischal Ranganath, Jack W McHugh, Supavit Chesdachai, Omar M Abu Saleh

**Affiliations:** Mayo Clinic Rochester, Rochester, Minnesota; Mayo Clinic, Rochester, Minnesota; Mayo Clinic, Rochester, Minnesota; Mayo Clinic, Rochester, Minnesota; Mayo Clinic Rochester, Rochester, Minnesota

## Abstract

**Background:**

*Achromobacter* spp. are important pathogens that are most frequently implicated in opportunistic infections, especially in patients with chronic lung disease, malignancy, and solid organ or hematopoietic stem cell transplantations. They commonly cause pneumonia, bacteremia, and device-associated infections. Intrinsic and acquired resistance mechanisms usually complicate treatment. Further characterization of the changing antibiotic susceptibilities and clinical manifestations of *Achromobacter* spp. are needed to improve clinical practice.

**Methods:**

We performed a retrospective review of *Achromobacter* isolates from 2013 to 2023 in the Mayo Clinic Enterprise with available antimicrobial susceptibility testing (AST) data. Variables included sample source, collection date, and AST by agar dilution with susceptibility defined according to Clinical and Laboratory Standards Institute (CLSI) breakpoints. Samples were categorized into quartiles based on collection date and grouped into source types (respiratory, genitourinary, etc.) and AST.

**Results:**

A total of 1,506 patients were identified with a total of 1,546 unique Achromobacter spp. isolates from 2013 - 2023. The most common species was A. xylosoxidans, encompassing 40.1% of all isolates (Table 1). Respiratory samples (sputum, bronchoalveolar lavage) were the most common source (57.5%), followed by musculoskeletal and soft tissue (16.4%), genitourinary (12.2%), and oropharyngeal sources (10.3%). AST from 2013 to 2023 showed persistent high levels of resistance to aztreonam and tetracycline, with stable susceptibility to piperacillin-tazobactam, trimethoprim-sulfamethoxazole, and carbapenems (Table 2). Over our 10-year analysis, there was increased resistance to aminoglycosides, fluoroquinolones, ceftazidime, and cefepime.

**Table 1.**
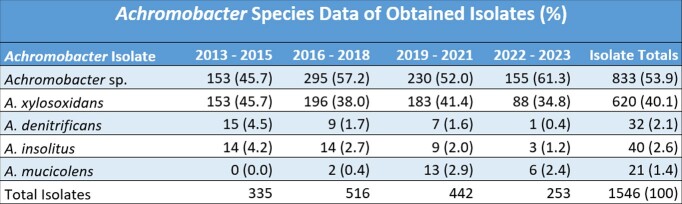
Achromobacter Species Identification and Isolate Percentage (%) Stratified by Year

Table 1 lists the Achromobacter species identified stratified by the year each sample was collected. Each value represents the number of isolates that were identified as the listed Achromobacter species, with "Achromobacter sp." indicating an isolate without species identification. Percentages of species are listed in parentheses (%). Sample totals are listed in the right column.

**Table 2.**
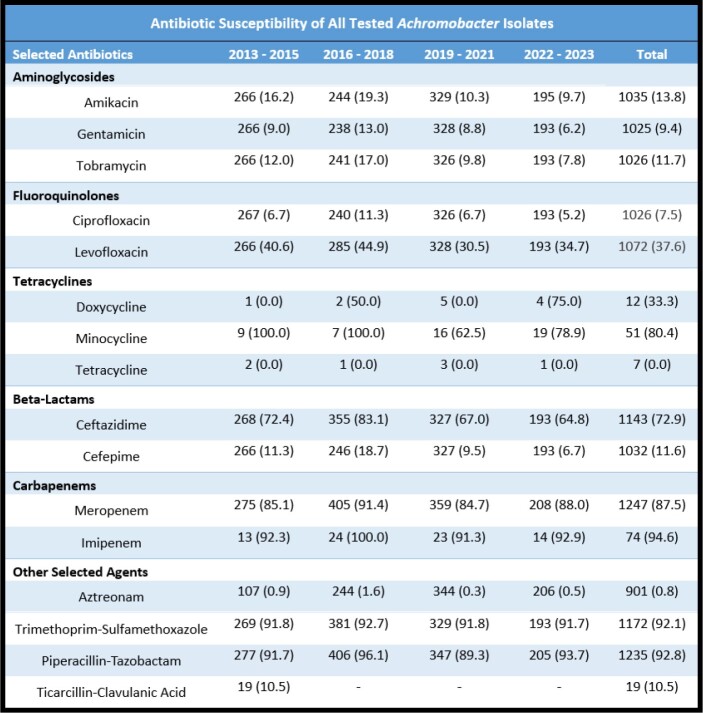
Antibiotic Susceptibility of All Tested Achromobacter Isolates (Percentage of Susceptible Isolates)

Table 2 lists antibiotics by class, stratified by year that Achromobacter isolates were tested. Each value is the total number of isolates tested for antimicrobial susceptibility, followed by the percentage of susceptible isolates in parentheses (%). Total isolates tested and overall susceptibility percentage is also listed over the 10-year period.

**Conclusion:**

*Achomobacter* continues to be a clinically significant organism with a complex resistance profile. Our dataset shows a significant prevalence of non-respiratory and non-genitourinary samples, in addition to increasing resistance over many antibiotic classes. Our data suggests that piperacillin-tazobactam, imipenem, and minocycline are appropriate empiric regiments for *Achromobacter*.

**Disclosures:**

**All Authors**: No reported disclosures

